# Complete Genome Analysis of an Enterovirus EV-B83 Isolated in China

**DOI:** 10.1038/srep29432

**Published:** 2016-07-12

**Authors:** Jingjing Tang, Qiongfen Li, Bingjun Tian, Jie Zhang, Kai Li, Zhengrong Ding, Lin Lu

**Affiliations:** 1 Yunnan Center for Disease Control and Prevention, Kunming, Yunnan Province, People’s Republic of China

## Abstract

Enterovirus B83 (EV-B83) is a recently identified member of enterovirus species B. It is a rarely reported serotype and up to date, only the complete genome sequence of the prototype strain from the United States is available. In this study, we describe the complete genomic characterization of an EV-B83 strain 246/YN/CHN/08HC isolated from a healthy child living in border region of Yunnan Province, China in 2008. Compared with the prototype strain, it had 79.6% similarity in the complete genome and 78.9% similarity in the *VP1* coding region, reflecting the great genetic divergence among them. *VP1*-coding region alignment revealed it had 77.2–91.3% with other EV-B83 sequences available in GenBank. Similarity plot analysis revealed it had higher identity with several other EV-B serotypes than the EV-B83 prototype strain in the *P2* and *P3* coding region, suggesting multiple recombination events might have occurred. The great genetic divergence with previously isolated strains and the extremely rare isolation suggest this serotype has circulated at a low epidemic strength for many years. This is the first report of complete genome of EV-B83 in China.

Human enteroviruses (EVs), comprising more than 100 serotypes, belong to the genus *Enterovirus*, family *Picornaviridae*^1^. They are among the most common human viruses, infecting an estimated number of a billion people annually worldwide^2^. Even though the majority of infections are often asymptomatic and goes unnoticed, these viruses are also associated with a variety of clinical presentations from minor febrile illness to severe conditions such as acute flaccid paralysis (AFP), meningitis, encephalitis, cardiac disease, pleurodynia, acute hemorrhagic conjunctivitis, hand-foot-and-mouth disease (HFMD), etc^3^. Each year, an estimated 10 to 15 million symptomatic EV infections occur in the United States^4^.

Enteroviruses are small, nonenveloped, single-stranded RNA viruses. EV genome is approximately 7.5 kb long and encodes a polyprotein that is processed to yield the mature structural (*VP4*, *VP2*, *VP3*, and *VP1*) and nonstructural (*2A, 2B, 2C, 3A, 3B, 3C*, and *3D*) proteins. The coding region is bounded by 5′ and 3′ untranslated regions (UTR)^3^.

Traditional EV serotypes, such as polioviruses, echoviruses, and group A and B coxsackieviruses, were identified via serological method about 50 years ago. There were several decades where new enteroviruses were uncommonly identified until the introduction of molecular detection methods in 1999^5^,^6^. In this system, members within an EV serotype share greater than 75% nucleotide (85% amino acid) identity in the *VP1* coding region. Then the last 16 years have seen a rapid expansion in the number of recognized enteroviruses and this period of discovery is still in progress^3^. The human enteroviruses are now classified into four species: Enterovirus A (EV-A), EV-B, EV-C, and EV-D^3^. EV-B has the most members (60 serotypes)^1^. Enterovirus B83 (EV-B83) is a newly identified EV-B type via molecular method. The prototype strain, isolated in the United States in 1976, was identified and reported in 2007^7^. Subsequently, several other EV-B83 strains were reported to be isolated from AFP cases or nonhuman primates in Bangladesh^8^,^9^, India^10^, and France^11^. Also in a previous study^12^, we reported the identification of two EV-B83 strains isolated in Yunnan Province of China in 1999 via sequencing the partial *VP1* region. However, to date, only the complete genome sequence of the prototype strain has been reported^7^.

In China, there is no specific case-based EV surveillance system, such as the National Enterovirus Surveillance System (NESS) in the United States. So, the AFP surveillance, designated for the global polio eradication, turns out to be an important approach in understanding the circulating enteroviruses in the population. Previous studies in the provinces of Yunnan and Shandong of China has described the serotype distribution and molecular epidemiology of non-polio enteroviruses (NPEV)^12^,^13^,^14^. Moreover, based on the AFP surveillance, several newer EV types have been identified and the complete genome has been sequenced and analyzed in China^15^,^16^,^17^,^18^,^19^.

Yunnan Province is located in southwest China bordering Myanmar, Vietnam, and Laos. The length of border line is 4060 kilometers, accounting for about 1/5 of China’s land border lines. Monitoring the importation of medical pathogens is of great public health importance. Therefore in addition to the AFP surveillance, we have carried on investigation about prevalence and distribution of human enteroviruses among healthy children in border area since 2007. In a previous study, we reported the identification and complete genome analysis of a new EV type EV-B106^16^. In this study, we describe the identification and genomic characterization of another new type EV-B83.

## Results

### Isolation and typing

The isolate 246/YN/CHN/08HC (abbreviated as YN08HC246) was recovered on both RD and HEp-2 cells. It cannot produce CPE in L20B cell line. Initially, serotyping was performed using the standard pools of EV serotyping antisera distributed by the World Health Organization. However, it could be neutralized by any pool of the antisera designated for most traditional EV serotypes. Then, *VP1* sequencing and molecular typing by the online genotyping tool^20^ indicated that the type of this isolate is EV-B83.

### *VP1* sequence analysis

Complete *VP1*-coding region alignment revealed strain YN08HC246 had 78.9% nucleotide and 95.4% amino acid similarities with EV-B83 prototype strain USA/CA76-10392. Since only partial *VP1* sequence of two previously isolated Yunnan EV-B83 strains can be obtained, it had 78.5% nucleotide and 98.1% amino acid similarities with previous Yunnan strain 146–99 based on alignment of 326-nt partial *VP1* coding region, and 80.9% nucleotide and 98.8% amino acid with previous Yunnan strain 159-99 based on 778-nt partial *VP1* coding region.

**Figure 1:**
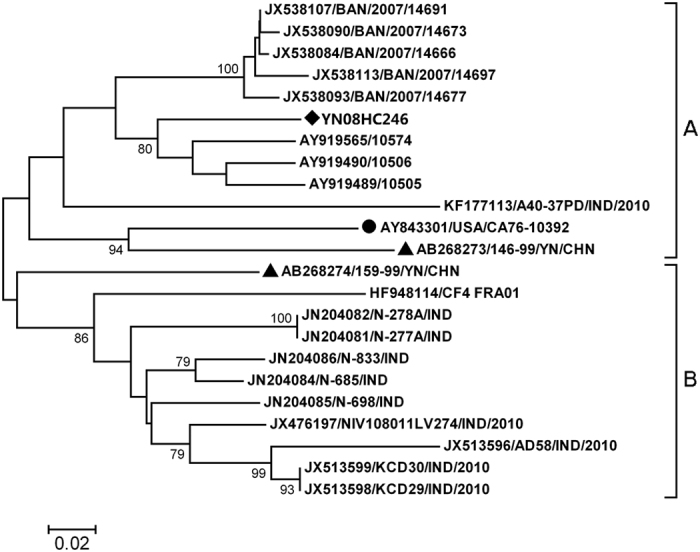
Phylogenetic tree based on partial *VP1* sequences of global EV-B83 isolates. The circle indicates the prototype strain, the diamond indicates EV-B83 isolate in this study, and triangles indicate previous Yunnan isolates. The tree is constructed based on the 255-nt (nucleotide position 2540–2794) partial *VP1* sequence.

There are altogether 22 EV-B83 *VP1* sequences in GenBank database that can be used in sequence analysis, whereas only eight are sequences of entire *VP1* coding region. Hence, sequence analysis was carried out based on 255-nt (nucleotide position 2540–2794) partial *VP1* coding region. In alignment with all 22 EV-B83 *VP1* sequences available in GenBank, strain YN08HC246 had 77.2–91.3% similarities with them. Phylogenetic analysis based on 255-nt partial *VP1* coding regions showed that two major clusters (A and B) were observed for global EV-B83 strains (Fig. 1). Strain YN08HC246 was segregated into cluster A which consisted of strains mostly from Bangladesh, alongside with a previous Yunnan strain, an Indian strain, and the prototype strain. The cluster B consisted of strains mostly from India, alongside with a previous Yunnan strain and a strain from France.

The within group means of *p*-distance for cluster A and B was 0.148 and 0.127, respectively, and the means of *p*-distance between the two clusters was 0.214. Strain YN08HC246 has an average *p*-distance of 0.180 with the others, suggesting great genetic divergence among them. Although both two previous Yunnan isolates, 146-99 and 159-99 were obtained in the same year, great genetic distance (0.224) was observed between them, suggesting the existence of at least two transmission chains at that time.

### Complete genome analysis

The complete genome of the strain YN08HC246 consisted of 7394 nucleotides. A 6552-nt open reading frame (ORF) encoded a potential polyprotein precursor of 2183 amino acid. The 5′ and 3′ *UTRs* consisted of 742 nt and 99 nt, respectively. Compared with the prototype strain, it had 79.6% similarity in the complete genome and 95.9% similarity in the deduced amino acid sequence of the potential polyprotein precursor. No nucleotide deletions or insertions were observed in alignment of strain YN08HC246 and the prototype strain. Strain YN08HC246 shared 79.7%, 80.3%, and 78.3% nucleotide identities in the *P1, P2,* and *P3* coding region respectively with the EV-B83 prototype strain, and 62.2–70.7%, 78.1–84.3%, and 77.4–85.0% nucleotide identities in the *P1, P2,* and *P3* region respectively with the other EV-B types. A comprehensive comparison on different genomic regions of the nucleotide sequence and deduced amino acid sequence of strain YN08HC246 with the EV-B83 prototype strain and other prototype strains belonging to EV-B is shown in Table 1.

### Phylogenetic analysis on *P1*, *P2* and *P3* coding regions

**Figure 2:**
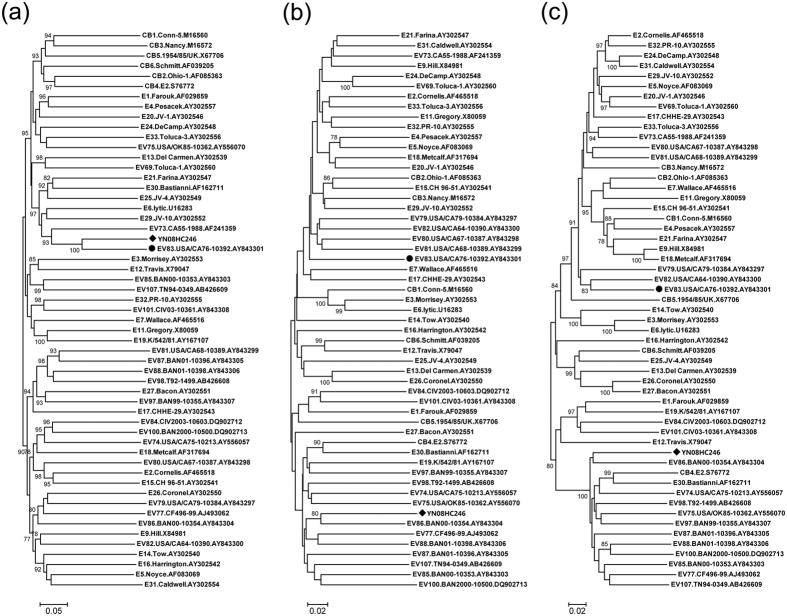
Phylogenetic relationships of the EV-B83 strain YN08HC246 and other EV-B prototype strains. The phylogenetic trees based on *P1* (**a**), *P2* (**b**), and *P3* (**c**) coding regions were constructed from the nucleotide sequence alignment using the neighbor-joining algorithm of the MEGA 5.0 software. The circle indicates the EV-B83 prototype strain, and the diamond indicates the EV-B83 isolate in this study.

Phylogenetic analysis of strain YN08HC246 and all EV-B prototype strains available in GenBank database was performed based on the *P1*, *P2*, and *P3* coding regions respectively (Fig. 2). In the *P1* capsid coding region, the Chinese strain YN08HC246 clustered together with EV-B83 prototype strain USA/CA76-10392 (Fig. 2a), consistent with the preliminary molecular typing results. The phylogeny in the non-structural protein regions turned out to be different (Fig. 2b,c). In the tree based on *P2* sequences, strain YN08HC246 clustered together with prototype strains of EV-B79 – EV-B82. In the tree based on *P3* sequences, strain YN08HC246 clustered together with EV-B82 prototype strain USA/CA64-10390. These results suggested the occurrence of one or more putative recombination events between EV-B83 strain YN08HC246 and other EV-B types.

### Recombination analysis with closely related strains

Recombination events usually occur in non-structural coding regions of the same species^21^,^22^,^23^. Since no close genetic relationship was observed between strain YN08HC246 and other EV-B prototypes based on the phylogenetic analysis in *P2* and *P3* coding regions (Fig. 2b,c) or homologous comparison on different genomic regions (Table 1), closely related sequences with strain YN08HC246 were screened using BLAST online. The *P2* and *P3* regions of strain YN08HC246 were separately analyzed using BLAST, and all sequences with high similarities were used in the recombination analysis.

**Figure 3:**
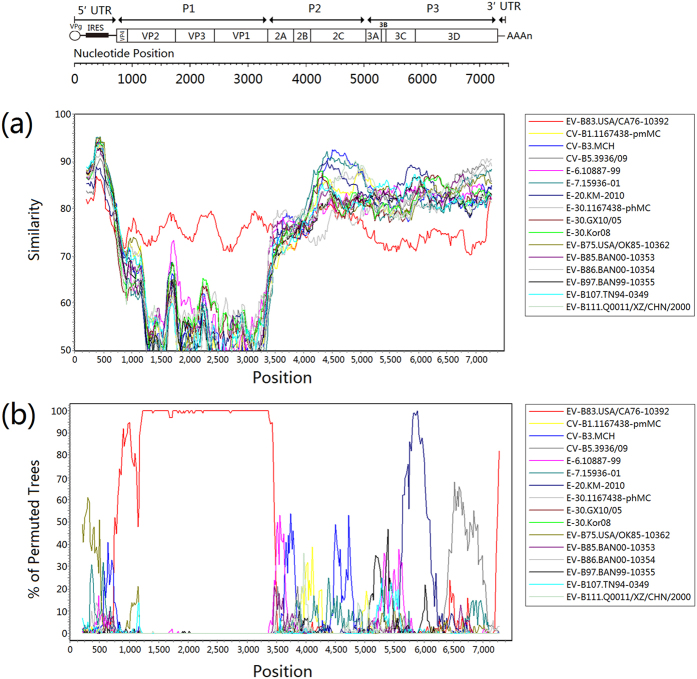
Similarity plot (**a**) and bootscanning analysis (**b**) of the Chinese EV-B83 strain with closely related strains. The analysis was conducted via Simplot v3.5.1 using a sliding window of 400 nucleotides moving in steps of 20 nucleotides. The genome of strain YN08HC246 serves as a query sequence. Multiple recombination events are suggested in *P2* and *P3* coding regions.

The similarity plot analysis revealed multiple recombination events in the genomic sequence of strain YN08HC246, and bootscanning analysis confirmed these recombination events (Fig. 3). In the *P1* coding region, strain YN08HC246 had the highest identity with the EV-B83 prototype strain. While in the *2A* and *2C* coding region, it had the highest similarities with E-6 strain 10887-99 and CV-B3 strain MCH, respectively. In the *3C* and *3D* coding region, it had the highest similarity with E-20 strain KM-2010. Bootscanning analysis confirmed the existence of recombination events between the Chinese EV-B83 strain and the related viruses.

## Discussion

In 1999, Oberste *et al.*^5^ introduced the molecular typing method for the identification of enterovirus isolates, based on reverse transcription–polymerase chain reaction (RT-PCR) amplification and sequencing of *VP1* region. This method has made it possible to identify new enterovirus serotypes, and has been widely adopted throughout the world. EV-B73 was the first new enterovirus serotype which had been previously deemed “untypeable” by serological identification method^24^. Subsequently, more and more new enterovirus types have been identified^7^,^25^,^26^,^27^,^28^. In China, the AFP surveillance has been established since 1994 for the global polio eradication, and in recent years, EV surveillance has been carried out in healthy children in some regions such as Xinjiang Uighur Autonomous Region^29^, Shenzhen City^30^, and so on. So, many NPEV isolates have been obtained in provincial polio labs. Molecular typing on these isolates will provide the molecular epidemiology of different enterovirus serotypes, and some new types have been reported in the provinces of Yunnan and Shandong recently^12^,^13^,^14^. In this study, we report the complete genome sequence of a new type, EV-B83.

EV-B83 is a rarely reported serotype. To date, there are limited EV-B83 nucleotide sequences available in GenBank nucleotide database. Our research teams have examined the NPEVs in Yunnan Province in the past ten years^12^,^13^. Altogether 293 NPEV isolates were obtained, but there were only 3 EV-B83 isolates. According to a retrospective investigation on NPEV in Shandong Province of China from 1988 to 2013^14^, a total of 962 NPEV isolates were obtained while no EV-B83 was identified. The limited detection in China and in the world suggests EV-B83 circulated at a low epidemic strength. However, the *VP1* sequence analysis in this study showed that great genetic divergence of more that 20% existed in the pairwise comparison of current EV-B83 isolates, suggesting near-saturation in allowable substitution within a type, since 75% identity is the cut-off value for type identity. So, it is reasonable to conclude that this serotype is not a newly emergent virus and has been circulated at a low epidemic level for many years.

To date, all EV-B83 strains deposited in the GenBank database come from five countries of the United States, Bangladesh, India, France, and China, in which two South Asian countries, Bangladesh and India, accounted for the majority (86%) of total isolates. Yunnan is a frontier province located in the southwest of China. It has frequent flow of the population with neighboring Southeast or South Asian countries. Previously, incidences of wild poliovirus importation from Myanmar to Yunnan occurred in 1995 and 1996^31^, and an importation of vaccine derived poliovirus from Myanmar occurred in 2012. Also, the previously reported EV-B106 strain was isolated from an AFP case in a region that borders Vietnam^16^. Hence, the surveillance for importation of new enterovirus serotypes or other medical pathogens in Yunnan Province should be of great concern. In this study, the EV-B83 strain was isolated from a 6 years old healthy child living in Lancang County bordering Myanmar. There is frequent population flow over among the border residents of the two countries, so the possibility of virus transmitting from Myanmar could not be ruled out.

EV-B83 prototype strain USA/CA76-10392 was isolated in California of the United States by inoculation of human fetal diploid kidney and primary monkey kidney cells^32^, but the information on its association with disease is not available. Due to the limited number of EV-B83 isolates in the world, its epidemiological data is scarce, so the biological and pathogenic properties of EV-B83 are currently difficult to study in detail. Some reported EV-B83 strains were isolated from stool samples of AFP cases, but no other data can be collected to conclude that EV-B83 can be causative agent of AFP. So, except for the potential association with paralysis, its pathogenic profile is far from well known. Further surveillance data might provide valuable information to understand whether this new type is associated with particular diseases or whether the transmission and circulation pathways differ in key ways from those of the known and well characterized serotypes.

Recombination within the nonstructural proteins among enteroviruses of the same species has been widely reported^33^,^34^,^35^. In this study, via phylogenetic analysis on *P1*, *P2,* and *P3* region, the phylogeny of the Chinese strain correlated with the type only in the *P1* region. No correlation was found, however, between sequence similarities with serotype in *P2* and *P3* regions, suggesting recombination in the noncapsid regions. As recombination usually occurred among enteroviruses within in a species, all available EV-B genomes should be included into the analysis. However, since the prototype strains have been isolated several decades ago, it is hard to detect exact recombination partners in comparing currently circulating strains with the prototype strains. So, as mentioned in the results section, we performed a preliminary screening for strains possessing high similarity with the Chinese EV-B83 strain in different coding regions via BLAST, and proposed recombination events were observed between strain YN08HC246 and E-6 strain 10887-99, CV-B3 strain MCH, and E-20 strain KM-2010 in different coding regions. Among them, E-6 strain 10887-99 was isolated from a patient with facial nerve paresis from Russia in 1999^36^; CV-B3 strain MCH was isolated from a case of fatal myocarditis in a newborn infant from the United States, and the virus was a genomic chimera that likely arose from recombination between coxsackievirus B3 and two newer types, EV-B86 and EV-B97^37^; E-20 strain KM-2010 was isolated from a patients infected by hepatitis A virus from Yunnan Province of China^38^. The similarity was still not as high enough to conclude these viruses as the exact recombination partner. However, the fact that the isolation sites of these isolates are geographically remote with each other suggests that long-term transmissions of these viruses have taken place, so as to provide the spatial and temporal circumstances for recombination to occur. Continuous and extensive surveillance and more genome data are needed to further understand the circulation and recombination of enteroviruses. In the study, although the recombinant EV-B83 strain was isolated from a healthy child, considering the high inapparent infection rate of human enteroviruses, and the observation of close genetic relationship in nonstructural regions with other neurovirulent enteroviruses, its pathogenicity should not be underestimated and its potential association with disease needs future epidemiological surveillance and case investigation.

In conclusion, this is the first report on the complete genome of EV-B83 in China. The great genetic divergence with previously isolated strains and the extremely rare isolation suggest this serotype has circulated at a low epidemic strength for many years, and multiple recombination events occurred in its evolution.

## Methods

### Ethics statement

This study did not involve human participants or experimentation. The only human material used was stool samples collected from a healthy child at the instigation of the Ministry of Health P. R. of China for public health purposes. Written informed consent for the use of the clinical samples was obtained from the legal guardian of the child. Ethical approval was given by the Ethics Review Committee of the Yunnan Center for Disease Control and Prevention, and the study was conducted in compliance with the principles of the Declaration of Helsinki.

### Virus isolation and initial serotyping

The strain YN08HC246 was recovered from a healthy child in Yunnan Province, China, in 2008. The child was a 6-year-old boy in the Lancang County bordering Myanmar.

Stool samples were collected and processed according to standard procedures recommended by the World Health Organization (WHO)^39^. Three cell lines, human rhabdomyosarcoma (RD), human laryngeal epidermoid carcinoma (HEp-2), and a mouse cell line carrying the human poliovirus receptor (L20B) cell lines were used for EV isolation. Two hundred microlitre of treated stool solution was added to each vial of the standard monolayer cell culture. The inoculated cells were examined every day. After 7 days, the tubes were frozen, thawed, re-passaged, and another 7 days examination was performed. Cell cultures with EV-like cytopathic effects (CPE) were harvested and used for further identification. To ensure no cross contamination had occurred, tubes of normal cells served as negative controls.

The micro-neutralization assays were carried out in 96-well tissue culture plates using enterovirus antiserum pools A to G against the most frequently isolated echoviruses and group B coxsackievirus. (National Institute for Public Health and the Environment, RIVM, the Netherlands).

### *VP1* RT-PCR, sequencing, and typing

Viral RNA was extracted from 140 μL of the infected cell culture by using the QIAamp Viral RNA Mini Kit (Qiagen, USA). Primer pairs 490–492^8^ and 187–011^5^ were used for amplifying the 5′ and 3′ part of the *VP1* coding region separately, and the combination of the two segments yields the entire *VP1* coding region. RT-PCR was performed by using the SuperScript III One-step RT-PCR System with Platinum Taq (Invitrogen, USA) according to the manufacturer’s instructions. In order to detect cross contamination, blank control and negative control were included in the RT-PCR reaction. PCR products were purified via QIAquick Gel Extraction Kit (Qiagen, USA), sequenced bi-directionally using the BigDye Terminator v3.0 Cycle Sequencing Kit, and analyzed using an ABI 3130 Genetic Analyzer (Applied Biosystems, Japan). The *VP1* coding region of the strain YN08HC246 were used for molecular typing by using online Enterovirus Genotyping Tool version 0.1^20^.

### Whole genomic sequencing

Four pairs of primers were used for amplification and sequencing of the rest of the genome (Table 2). The 5′ and 3′ end sequence of the genome of strain YN08HC246 was obtained using the 5′/3′ RACE Kit (Roche, German) according to the recommended procedure. Primers A812, A576, and A408 were used to amplify the 5′ end sequence. Primer S6945 was used to amplify the 3′ end sequence. All these primers used in the whole genomic sequencing were designated in this study. Positive products were purified and bi-directionally sequenced as described above.

### Sequence analysis and recombination analysis

Nucleotide and amino acid sequence alignment was performed by using the BioEdit (version 7.2.3)^40^. Phylogenetic analysis was conducted via using MEGA version 5.0^41^ using the neighbor-joining method with a Kimura two-parameter model. Bootstrapping was performed with 1000 duplicates and bootstrap values greater than 75% were considered statistically significant for grouping. Similarity plot and bootscanning analysis was performed by using the Simplot 3.5.1 program with a 400 nucleotide window moving in 20 nt steps and a Jukes–Cantor correction^42^,^43^. The EV-B83 prototype strain USA/CA76-10392 and other closely related sequences^44^,^45^,^46^,^47^,^48^ used in the recombination analysis were listed in Table 3.

### Nucleotide sequence accession number

The complete genome sequence of the EV-B83 strain YN08HC246 described in this study was deposited in the GenBank database under the accession number KU707902.

## Additional Information

**How to cite this article**: Tang, J. *et al.* Complete Genome Analysis of an Enterovirus EV-B83 Isolated in China. *Sci. Rep.* **6**, 29432; doi: 10.1038/srep29432 (2016).

## Figures and Tables

**Table 1:** Nucleotide and deduced amino acid identities of strain YN08HC246 with EV-B83 prototype strain USA/CA76-10392 and other EV-B prototype strains.

Region	Identity with USA/CA76-10392 (%)		Identity with other EV-B (%)	
	Nucleotide	Amino acid	Nucleotide	Amino acid
*5′ UTR*	81.2	/	77.7–87.2	/
*VP4*	80.1	91.3	67.1–79.2	73.9–92.7
*VP2*	79.7	97.6	65.1–71.8	73.6–84.2
*VP3*	80.3	98.3	61.5–73.6	67.7–83.1
*VP1*	78.9	95.4	53.8–68.0	56.6–71.2
*2A*	78.2	94.0	74.0–82.0	88.0–94.6
*2B*	77.1	94.9	75.4–82.1	91.9–97.9
*2C*	82.3	96.9	79.1–86.5	95.7–99.0
*3A*	78.2	95.5	75.6–85.7	93.2–98.8
*3B*	75.7	95.4	72.7–92.4	86.3–95.4
*3C*	77.4	95.0	76.5–85.6	92.3–98.3
*3D*	78.7	95.2	77.0–86.2	94.3–98.7
*3′ UTR*	92.1	/	80.3–93.1	/

**Table 2:** Primers used for complete genome amplification and sequencing.

Primer	Sequence (5′-3′)	Nucleotide position*	Orientation
1S	TTAAAACAGCCTGTGGGTTGWWCCCACCCAC	1–31	Forward
546A	GAAACACGGACACCCAAAGTA	566–546	Reverse
455S	CCCCTGAATGCGGCTAATCC	455–474	Forward
2555A	AACGTGTCTCGTCTGCATGGTGTCACT	2581–2555	Reverse
3219S	AGGCTGTTACCGATCAACGCGGCGACCAT	3219–3247	Forward
5594A	GATGTCYCTRAAYTTYTCRTT	5614–5594	Reverse
5366S	TTTGARTTYGCIGTIGCIATGATGAA	5366–5391	Forward
7370A	CCGCACCGAATGCGGAGAATTTACC	7394–7370	Reverse
A812^#^	TATGTTTGTATAGTGGATAAT	832–812	Reverse
A576^#^	ACCATAAGCAGCCAGTGTAA	595–576	Reverse
A408^#^	ACTCTTCGCACCATGTCGGT	427–408	Reverse
S6945^#^	CGGGGAAAGGGTATGGTCTGATTAT	6945–6969	Forward

^*^Numbering according to the genome of EV-B83 prototype strain USA/CA76-10392.

Primers marked with “#” are only used for 5′ and 3′ RACE.

**Table 3:** Information on the EV-B83 prototype strain USA/CA76-10392 and other closely related sequences used in the recombination analysis.

Type	Strain	Country	Year	Accession number
EV-B83	USA/CA76-10392	United States	1976	AY843301
CV-B1	1167438-pmMC	Switzerland	2010	JN797615
CV-B3	MCH	United States	2005	EU144042
CV-B5	Sep-36	Kuwait	2009	KP233830
E-6	10887-99	Russia	1999	AY896760
E-7	15936-01	Azerbaijan	2001	AY896765
E-20	KM-2010	China	2010	KF812551
E-30	1167438-phMC	Switzerland	2009	JN797616
E-30	GX10/05	China	2010	JX854435
E-30	Kor08	South Korea	2008	JN704615
EV-B75	USA/OK85-10362	United States	1985	AY556070
EV-B85	BAN00-10353	Bangladesh	2000	AY843303
EV-B86	BAN00-10354	Bangladesh	2000	AY843304
EV-B97	BAN99-10355	Bangladesh	1999	BAN99-10355
EV-B107	TN94-0349	Japan	1994	AB426609
EV-B111	Q0011/XZ/CHN/2000	China	2000	KF312882
